# A puromycin selectable cell line for the enrichment of mouse embryonic stem cell-derived V3 interneurons

**DOI:** 10.1186/s13287-015-0213-z

**Published:** 2015-11-10

**Authors:** Hao Xu, Nisha Iyer, James E. Huettner, Shelly E. Sakiyama-Elbert

**Affiliations:** Department of Biomedical Engineering, Washington University in St. Louis, 1 Brookings Drive, Box 1097, St. Louis, MO 63130 USA; Department of Cell Biology and Physiology, Washington University School of Medicine, St. Louis, MO USA

**Keywords:** Electrophysiology, Functional neuronal maturation, Neural differentiation, Recombineering, Sim1, Spinal interneuron

## Abstract

**Introduction:**

Spinal V3 interneurons (INs) are a commissural, glutamatergic, propriospinal neuron population that holds great potential for understanding locomotion circuitry and local rewiring after spinal cord injury. Embryonic stem cells hold promise as a cell source. However, the inevitable heterogeneity resulting from differentiation protocols makes studying post-mitotic stem cell-derived neuron populations difficult because proliferative glia quickly overtake a culture. Previously, an induction protocol for V3 INs was established. However, because of the heterogeneous population resulting from the induction protocol, functional characterization of the induced cells was not possible.

**Methods:**

A selectable murine transgenic embryonic stem cell (ESC) line (Sim1-Puro) was generated by recombineering. The expression of the puromycin resistance enzyme, puromycin N-acetyl-transferase (PAC), was knocked into the locus of a post-mitotic V3 IN marker (Sim1), allowing Sim1 gene regulatory elements to control PAC expression. The resulting cell line was characterized for Sim1 expression by in situ hybridization, for glutamatergic marker expression by immunocytochemistry and quantitative real time polymerase chain reaction (qRT-PCR), and for functional maturation by electrophysiology.

**Results:**

Puromycin selection significantly enriched the population for V3 INs, allowing long-term characterization. The selected population expressed the neuronal marker β-III tubulin and the glutamatergic neuron marker VGluT2. The selected V3 INs also exhibited appropriate functional maturation, as assessed by electrophysiology, and remained glutamatergic for 2 weeks.

**Conclusion:**

The Sim1-Puro cell line provides a simple, high throughput method for generating large numbers of V3 INs from mouse ESCs for future in vitro and cell transplantation studies.

**Electronic supplementary material:**

The online version of this article (doi:10.1186/s13287-015-0213-z) contains supplementary material, which is available to authorized users.

## Introduction

Pluripotent stem cells have the potential to be a plentiful cell source for many different cell types. Differentiation protocols have been established to generate specific cell types from embryonic stem cells (ESCs) and induced pluripotent stem cells. In the central nervous system, differentiation protocols for ESCs have been developed for several populations, including midbrain and hindbrain dopaminergic and serotonergic neurons [1, 2], spinal motoneurons (MNs) [3, 4], and V0, V2a, and V3 spinal interneurons (INs) [5–7]. These populations (MNs and INs) contribute to locomotion either by directly innervating muscle or by playing a role in central pattern generator (CPG) circuitry [8–12]. By deriving ventral spinal populations from ESCs or induced pluripotent stem cells, we can better study these populations in culture and their role in locomotion and reorganization after spinal cord injury.

One ventral spinal population that may contribute to reorganization after spinal cord injury is the commissural, glutamatergic V3 IN population. V3 INs arise from the Nkx2.2^+^ p3 progenitor domain and express the transcription factor Sim1 upon reaching the post-mitotic stage [13]. They contribute to rhythm generation networks within the spinal cord by playing a role in regulating left–right alternation of gait and in balancing locomotor outputs [12, 14]. This population has been shown to cross multiple spinal segments and synapse onto MNs and other INs [12, 14]. During maturation, V3 INs separate spatially during post-mitotic development and become recruited during running or swimming behaviors [14]. Their importance in locomotion makes V3 INs a key target to better understand locomotor coordination and a potential cell therapy candidate for functional recovery and local reorganization after spinal cord injury. Unfortunately, the isolation of primary V3 INs from mouse spinal cords is not technically feasible, driving the need for an in vitro method to generate them.

Neurons of the ventral spinal cord have been generated from mouse ESCs by exposing embryoid bodies (EBs) to sonic hedgehog (Shh) pathway agonists and retinoic acid (RA) [3, 4]. Shh forms a dorsal–ventral gradient in the neural tube during development and helps to establish the five ventral spinal progenitor domains, which then mature into post-mitotic spinal neurons [15–18]. RA from the lateral somites acts as a caudalizing factor to impart spinal identity [3, 19, 20]. The protocol to derive MNs from ESCs uses relatively high RA and Shh concentrations to induce spinal MNs [3, 4, 21]. To induce V3 INs, the p3 progenitor domain location was examined in relation to the progenitor MN domain location. Being a more ventral population, V3 INs sit closer to the floor plate and notochord, and therefore would be exposed to increased Shh signal strength and duration [22]. Additionally, being further away from the lateral somites, V3 INs would be exposed to lower RA concentrations. While V3 INs can be generated via induction of mouse ESCs, the yield is relatively low (~8 %) [7].

Unfortunately, ESC differentiation protocols generally yield heterogeneous populations, and this heterogeneity makes live cell identification and characterization difficult. Heterogeneity also negatively affects transplantation – often generating teratomas and causing regression after short-term improvements due to incomplete cell differentiation or maturation [23–25]. Some methods to reduce this heterogeneity include density-based centrifugation, fluorescence-activated cell sorting (FACS), and use of transgenic cell lines with antibiotic resistance. Density gradient-based MN separation protocols have been used to improve MN yield [26, 27]. However, V3 INs are similar in size to many other IN populations, making isolation of V3 INs from other spinal cells difficult. FACS requires either a unique cell surface marker or genetic engineering of a fluorescent marker to isolate pure populations [28], and it is slow for purification of cells that make up a small percentage of the initial population (<10 %) [29]. FACS also increases the risk for contaminated cultures and can result in low viability for post-mitotic neurons. Antibiotic resistance has a long history within biology for positive selection of desired traits and is much more scalable compared to FACS. Additionally, once an antibiotic resistant cell line is generated, no specialized equipment is required for cell isolation. Cell lines for antibiotic resistance have been previously utilized for the enrichment of neural lineage populations [30, 31]. Recently, our lab generated mouse ESC lines expressing puromycin-N-acetyltransferase (PAC, a puromycin resistance gene) for the positive selection of induced neural progenitors and neuronal populations [21, 32]. In these cell lines, PAC was expressed either under Olig2, a marker for progenitor MNs (pMNs), or Hb9, a marker for post-mitotic MNs. These cell lines utilized drug selection as a high throughput, low cost method to enrich cell populations for the desired cell type based on developmental marker expression. We hypothesized that incorporating PAC into the Sim1 locus would allow for the enrichment of a previously reported V3 IN induction via antibiotic selection. The selected cells should allow for the studying of V3 INs in vitro, including the confirmation of Sim1^+^ cells’ glutamatergic identity.

## Methods

### ESC culture

This work involved no animals and only ESCs and ESC-derived cell types. All ESCs were cultured on T-25 flasks coated in 0.1 % gelatin (Sigma, St. Louis, MO). Cells were cultured in complete media consisting of Dulbecco’s Modified Eagle Medium (DMEM 11965, Life Technologies, Carlsbad, CA) containing 10 % newborn calf serum (Life Technologies), 10 % fetal bovine serum (Life Technologies), and a 1:100 dilution of a 100× nucleoside mix (EMD Millipore, Bellerica, MA). Cells were routinely passaged by washing with DMEM 11965 containing 25 mM HEPES (Life Technologies), dissociating with 1 mL 0.25 % Trypsin Ethylenediaminetetraacetic acid (EDTA; Life Technologies), quenching with complete media, and plating into a new T-25 gelatin-coated flask containing a final volume of 5 mL media with 1000 U/mL leukemia inhibitory factor (EMD Millipore) and 100 μM β-mercaptoethanol (Life Technologies).

### Generation of Sim1-Puro-pStartTK targeting vector

**Fig. 1:**
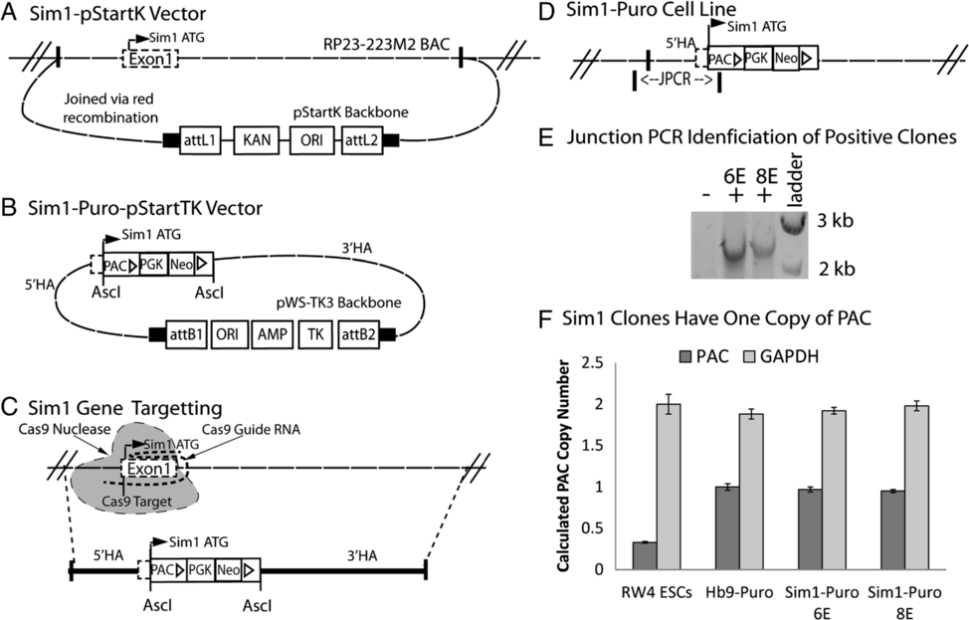
Generation and identification of Sim1-Puro cell line. **a** Red recombineering was utilized to insert the region approximately 2 kb upstream to 10 kb downstream of Exon 1 of the Sim1 gene from RP23-223 M2 BAC into the pStartK backbone, generating the Sim1-pStartK plasmid. **b** After AscI cut sites were introduced by another red recombination reaction, a PAC-PGK-Neo cassette was inserted into the open reading frame of Sim1 Exon1in the Sim1-pStartK plasmid. Using gateway recombination, the pStartK backbone was replaced with the pWS-TK3 backbone to introduce the negative selection gene, TK. 5’ and 3’ homology arms are labeled. **c** CRISPR/Cas9 targeting of the Sim1 gene was used to generate a double stranded break at the desired recombination location. Sim1-Puro-pStartTK recombined into the Sim1 locus as shown by the dotted lines. 5’ and 3’ homology arms are labeled. **d** The Sim1-Puro cell line with PAC in Sim1 Exon 1. Junction PCR (JPCR) primer for approximately 2.6 kb was used to screen for the desired recombination event. **e** JPCR bands of positive (+) and negative (−) clones with a 1 kb ladder. **f** Copy number assay shows Sim1 clones have one copy of PAC by comparison with RW4 ESCs (0 copy) and Hb9-Puro ESC (1 copy) controls. AMP, Ampicillin resistance gene; AscI, Restriction enzyme site; attB1 & attB2, Gateway recombination results; HA, Homology arm; JPCR, Junction PCR; ori, Origin of replication; PAC, Puromycin resistance gene; PGK, Phosphoglycerate kinase promoter sequence; Neo, Neomycin resistance gene; Sim1 ATG, Translation start in Sim1 Exon1; TK, Thymidine kinase

The *Sim1-Puro-pStartTK* targeting vector was constructed following a previously published protocol [33]. The backbone was a Gateway-compatible plasmid, pStartK (Addgene, Cambridge, MA). Sim1 homology arms were incorporated into pStartK from RP23-223 M2 BAC (BACPAC Resource Center, Children’s Hospital Oakland Research Institute, Oakland, CA) using pstartK_Sim1_upstream and pstartK_Sim1_downstream primers (Table 1) by recombineering techniques with red recombinase competent bacteria (Sim1-pStartK, Fig. 1a). A chloramphenicol resistance gene flanked by AscI cut sites from pkD3 (The E. Coli Genetic Stock Center, Yale University, New Haven, CT) was inserted into the open reading frame of the Sim1 gene by recombineering with primers Sim1_CAT_Forward and Sim1_CAT_Reverse 900 bp (Table 1). The chloramphenicol resistance gene was then replaced via restriction enzyme digestion and ligation by a dual resistance cassette consisting of, from 5’ to 3’: Asc1 cut site, Kozak sequence, PAC with bgh polyA signal, floxed phosphoglycerate kinase I promoter driving neomycin phosphotransferase (PGK-neo) with bgh polyA signal, and AscI site (gift from Dr. David Gottlieb, Washington University, St. Louis, MO) [21]. A negative selection thymidine kinase gene was incorporated into the finished vector (Sim1-Puro-pStartTK, Fig. 1b) using pWS-TK3 plasmid (Addgene) and Gateway LR clonase II kit (Life Technologies). For a more detailed diagrams of the recombineering steps, see Additional file 1: Figure S1.

**Table 1:** Primers for Sim1-Puro generation

Primer name	Sequence
pstartK_Sim1_upstream	AAAGTACTGTTTCTGGGGAAAACTCTAGTTTAGAGACCCTCCTGTTCTAAcgactgaattggttcctttaaagc
pstartK_Sim1_downstream	GAACCAGGCAGAGGGAAAGCTTCTCATTAGTGCTTTTCCCTTCTCTCTCCgccgcactcgagatatctagaccca
Sim1_CAT_Forward	GATGAGTCTGTGGAGTTTACGTTGTAAGAAGAAAGGGAGCCCGAGACACGGGCGCGCCagcattacacgtcttgagcgattgt
Sim1_CAT_Reverse 900 bp	TTGAGGAAGGGTGAGCAAATGGGAGATCAAAGAGCTCCTTCCCTGGAGAGGGCGCGCCcacttaacggctgacatgggaatta
Sim1_Fwd_Junction1	atgcacacgactcttcaaagaa
Puro_Reverse Junction1	gcgccaggaggccttccatctgttgct

Capital letters indicate homology arms for red recombination

### Generation of Sim1-Puro ESCs

The Sim1-Puro cell line was generated from the RW4 mouse ESC line (American Type Culture Collection, Manassas, VA). Approximately 1 × 10^7^ RW4 ESCs were resuspended in electroporation buffer with 10 μg of *Sim1-Puro-pStartTK* vector and 200–300 ng of a Cas9 guide RNA vector (deemed gSim1.MS8.mSim1.g6a, with guide RNA (Fig. 1c, Cas9 Guide RNA) targeting 5’-gtccatcattcgtgtcttcc cgg-3’ near the Sim1 start codon (Fig. 1c, Cas9 Target)) in the MLM3636 plasmid (Addgene plasmid #43860) and 200–300 ng of the Cas9 nuclease expression plasmid p3s-Cas9HC (Addgene plasmid #43945). Both Cas9 vectors were obtained from Genome Engineering Core, Washington University in St. Louis and originally gifted by Keith Joung and Jin-Soo Kim, respectively. Cells were electroporated using a Biorad Gene Pulser Xcell Eukaryotic System at 0.23 kV and 975 μF in a 0.4 cm cuvette (Bio-Rad, Hercules, CA). Following electroporation, cells were seeded on gelatin-coated 100 mm dishes for 24 hours then treated with G418 (200 μg/mL, Life Technologies) and 1-(2-Deoxy-2-fluoro-β-D-arabinofuranosyl)-5-iodouracil (150 nM, Movarek Biochemicals, Brea, CA) for positive and negative selection, respectively. After 14 days, surviving clones were picked and seeded into individual wells of a gelatin-coated 96 well plate.

### PCR screening on Sim1-Puro clones

Clones were screened for targeting events by junction polymerase chain reaction (JPCR, Fig. 1d). One primer binding outside of the left homology arm (5’ HA, Fig. 1d) and the other primer binding inside the PAC gene were used to screen for clones that properly incorporated the PAC gene. Reactions were performed using a Mastercycler Nexus Gradient thermocycler (Eppendorf, Hauppauge, NY) with primers Sim1_Fwd_Junction1 and Puro_Reverse Junction1 (Table 1 and Fig. 1d) at 95 °C for 60s, followed by 35 cycles of 94 °C for 20s, 60 °C for 30s, and 72 °C for 120 s.

### Copy number assay

Taqman Copy number assay (Life Technologies) was performed on cell lysates as per manufacturer instructions. Gapdh (Mm00186825_cn, Life Technologies) was normalized to RW4 ESCs, and PAC (custom ordered PAC assay, Life Technologies) was normalized to a previously published Hb9-Puro cell line [32]. Analysis was performed using Life Technologies CopyCaller v2.0.

### V3 IN induction

**Fig. 2:**
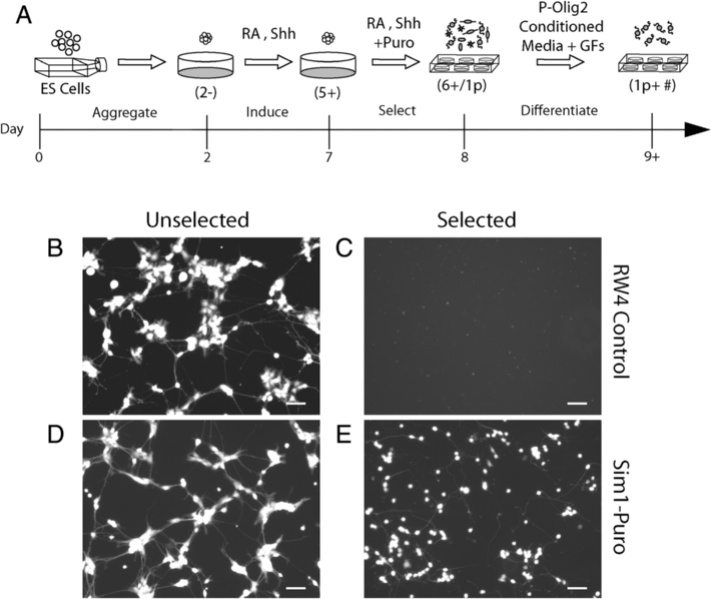
The effect of selection on survival of RW4 and Sim1-Puro embryonic stem cells (ESCs). **a** Diagram depicting induction and selection process. Induction and differentiation nomenclature is indicated by parenthesis while the time course (in days) is denoted by the time line. Briefly, ESCs are allowed to aggregate for 2 days (2^−^) to form embryoid bodies (EBs). EBs are induced with retinoic acid (RA) and sonic hedgehog (Shh) for 5 days (5^+^) and selected with puromycin in the presence of RA and Shh for 1 day (6^+^/ 1p). Selected cells are allowed to differentiate after selection (1p + #). **b** & **d** Live (calcein-AM) staining on unselected cells. **c** & **e** Live staining of selected cells. Scale bar = 50 μm

RW4 ESCs and Sim1-Puro ESCs were aggregated to form EBs on a non-adhesive agar-coated surface and induced to generate neural progenitors using our previously established 8-day induction protocol (2^−^/6^+^, where “2^−^” refers to the number of days ESCs are allowed to aggregate into EBs without (−) RA and smoothened agonist (SAG, a Shh pathway agonist) and “#^+^”refers to the number of days the EBs are exposed to (+) RA and SAG; Fig. 2a) [7]. Cells were cultured in suspension for 2 days on 100 mm Petri dishes pre-coated with 0.1 % agar (Thermo Fisher Scientific, Waltham, MA) in DFK-5 media comprised of 1:1 DMEM/F12 (Life Technologies) with 5 % knockout serum replacement (Life Technologies), 1× insulin transferrin selenium (Life Technologies), 100 μM β-mercaptoethanol, 50 μM non-essential amino acids (Life Technologies), and a 1:200 dilution of a 100× nucleoside mix. During the first 2 days, the cells aggregated into multi-cellular EBs. After aggregation, EBs were removed and allowed to settle. The supernatant was discarded and replaced with new DFK-5 media supplemented with 10 mM RA (Sigma) and 0.5 μM SAG (EMD, Millipore). Media was replaced every 2 days for 6 days.

### Selection and differentiation of V3 INs

To test PAC expression in Sim1^+^ cells, RW4 and Sim1-Puro EBs were subjected to induction and selection protocols as illustrated in Fig. 2a. On 2^−^/4^+^, standard tissue culture plates were pre-coated with 0.01 % poly(ornithine) solution (Sigma) at 37 °C for 1 hour. Poly(ornithine) was removed and plates were washed with a HEPES buffered saline solution three times. Plates were then coated with 0.01 mg/mL laminin (Life Technologies) in HEPES buffered saline solution overnight and washed three times before use as laminin coated plates. One day prior to the end of V3 IN induction (on 2^−^/5^+^), EBs were allowed to settle, and the DFK-5 media supernatant was collected. EBs were dissociated with 0.25 % trypsin-EDTA for 15 minutes at 37 °C, quenched with complete media, pelleted and replated at 3.5 × 10^7^ cells/cm^2^ onto laminin-coated plates. Cells were incubated with 2 μg/mL puromycin (Sigma) in the collected DFK-5 supernatant for 1 day (designated as 1p, Fig. 2a). After one day, puromycin-containing media was removed and replaced with “P-Olig2-conditioned” media (see next section) with supplemental factors (1× Glutamax (Life Technologies), and 5 ng/mL each of NT-3, GDNF, and BDNF (all from Peprotech, Rock Hill, NJ). Cells were allowed to differentiate up to 14 days (designed as “+ #” where # is the number of days after selection, Fig. 2a).

### Generation of P-Olig2-conditioned V3 media for long-term V3 cultures

After selection, cells were initially cultured in a 1:1 mix of DFK-5 and Neurobasal (Life Technologies) media with supplemental factors (listed above). However, due to the low cell density, extensive cell death was observed between 3 and 7 days post-selection (1p +3 and 1p +7). Thus, to improve cell survival, conditioned media generated by progenitor motor neuron cultures containing glia (oligodendrocyte and astrocytes) were used. A puromycin-selectable-pMN ESC line (P-Olig2), with PAC knocked into the Olig2 locus, underwent 2^−^/4^+^ induction using DFK-5 media with 2 μM RA and 0.5 μM SAG (2 days without and 4 days with RA/SAG) as previously described [21]. On the last 2 days of pMN induction (2^−^/2^+^ – 2^−^/4^+^), cells were selected with 4 μg/mL puromycin. On 2^−^/4^+^, the selected EBs were dissociated and plated at a density of 100,000 cells/mL in a 1:1 mix of DFK-5 and Neurobasal media with 1× B27 (Life Technologies) in a 6-well laminin-coated plate. Conditioned media was collected and replaced with fresh media every 2 days. Selected P-Olig2 cells were cultured up to 14 days.

### Live assay

Live reagent, calcein-AM (Life Technologies), was used to visualize live cells, respectively. Wells were washed with DMEM 11965 containing 25 mM HEPES and incubated with 0.325 μL/mL of 4 mM stock concentration calcein-AM (live) for 30 min at room temperature.

### Probe synthesis for in situ hybridization

Plasmids for in situ hybridization probes were a generous gift from Dr. Paul Gray (Washington University in St. Louis) [34]. Gene fragments from verified plasmids were linearized by direct PCR amplification using ReadyMade Primers (SP6 Promoter and T7 Promoter, Integrated DNA Technologies, Coralville, IA). Digoxigenin (DIG)-labeled antisense and sense RNA probes were made using PCR products as templates and T7 RNA polymerases (Roche, Indianapolis, IN). Probes were used at a concentration of 1–2 μg/mL. Sense counterparts of all probes were tested to ensure probe specificity.

### In situ hybridization

To assess the purity of cells post-selection, in situ hybridization was performed on dissociated cells. After 24 hours of selection, cultured cells were fixed in 4 % paraformaldehyde (PFA) for 10 minutes and washed three times in diethylpyrocarbonate-treated phosphate buffered saline (PBS; Sigma) at room temperature. Next, cells were incubated in 0.1 M triethanolamine-HCl (1.3 % triethanol amine (Sigma) and 0.4 % HCl (Thermo Fisher Scientific) with 0.25 % acetic anhydride (Sigma)) for 10 minutes. Cells were washed in 1× sodium citrate buffer for 5 minutes at room temperature and permeabilized in 0.2 M HCl in diethylpyrocarbonate-water for 10 minutes. Three additional washes with diethylpyrocarbonate-treated PBS were performed before cells were blocked in hybridization buffer (50 % formaldehyde (Sigma), 5× sodium citrate buffer (SSC, Life Technologies), 0.3 mg/mL yeast RNA (Sigma), 0.1 mg/mL heparin (Sigma), 1× Denhardt’s solution (Life Technologies), 0.1 % tween (Sigma), and 5 mM EDTA) for 4–6 hours at room temperature. Cells were incubated in hybridization buffer containing 1–2 μg/mL DIG-labeled antisense RNA probes (see previous section) overnight at 65 °C. Probed cells were washed twice in 0.2× SSC at 62 °C, and incubated in 0.2× SSC for 60 minutes at 65 °C. Washed cells were adjusted to room temperature and blocked with 10 % deactivated horse serum (Life Technologies) in PBS with 2 mg/mL bovine serum albumin and 0.1 % Triton X-100 (PBT) and incubated in alkaline phosphatase-labeled anti-DIG antibody (1:2000 in 10 % deactivated horse serum in PBT; Roche) overnight. Cells were further washed with PBT and color was visualized using nitro blue tetrazolium and 5-bromo-4-chloro-3-indolyl phosphate (Roche). Staining was stopped with 4 % PFA after visual inspection. Cell nuclei were stained with the nuclei binding dye Hoechst (1:1000, Life Technologies).

### Immunocytochemistry

Neuronal cell identity was assessed in differentiated cultures using immunocytochemistry. Cell cultures were fixed with 4 % PFA for 30 min then permeabilized in 0.01 % Triton X (Sigma) for 15 min. The cells were blocked with 5 % normal goat serum for 1 hour at 4 °C and incubated overnight at 4 °C in 2 % normal goat serum solution with one or more of the following primary antibodies: rabbit anti-Tuj1 (for β-III tubulin, 1:200, Covance, Princeton, NJ), guinea pig anti-VGluT2 (1:3000, EMD Millipore), mouse anti-SV2 (1:100, Developmental Studies Hybridoma Bank, Iowa City, IA), rabbit anti-MAP2 (1:1000, EMD Millipore), mouse anti-bassoon (1:600, Enzo Life Sciences, Farmingdale, NY), mouse anti-Isl1 (1:100, Developmental Studies Hybridoma Bank), mouse anti-Hb9 (1:20, Developmental Studies Hybridoma Bank), mouse anti-A2B5 (1:25, Developmental Studies Hybridoma Bank), mouse anti-nestin (1:100, Developmental Studies Hybridoma Bank), mouse anti-Nkx2.2 (1:100, Developmental Studies Hybridoma Bank), rabbit anti-Ki-67 (1:100, Abcam, Cambridge, MA), mouse anti-O4 (1:100, EMD Millipore), and mouse anti-Uncx (1:500, EMD Millipore). Primary antibody staining was followed by three washes in an excess volume of PBS for 15 min each. Each culture was then stained with appropriate Alexa Fluor secondary antibodies (1:1000, Life Technologies) for 1 hour at 4 °C followed by an additional three washes in PBS. Cell nuclei were stained with Hoechst (1:1000, Life Technologies).

### Image capture and analysis

All images were captured using a MICROfire camera (Olympus, Center Valley, PA) attached to an Olympus IX70 inverted microscope using either a 10× or 20× objective. Images were merged and colored using ImageJ (US National Institutes of Health, Bethesda, MA). To remove human bias, ImageJ was used for the automated counting of Hoechst-labeled nuclei. Images underwent thresholding to remove background and were then converted to binary black and white images. The “Analyze Particles” function was used to count the nuclei, excluding any small (less than 600 pixels) punctate nuclei to prevent apoptotic Hoechst^+^ nuclear debris from being counted.

### Quantitative real-time PCR (qRT-PCR)

Fourteen days after selection (1p +14), selected and unselected (control) cells on a 24-well plate were lysed with 350 μL of buffer RLT from the RNeasy Mini Kit (Qiagen, Valencia, CA). High Capacity RNA-to-cDNA Kit (Life Technologies) generated cDNA from RNA that was extracted per Qiagen’s instructions. TaqMan Fast Advanced Master Mix (Life Technologies) was combined with TaqMan Gene Expression Assays (Life Technologies; Table 2) and cDNA for qRT-PCR. Reactions were performed using a Step One Plus Applied Biosystems thermocycler (Life Techonologies) with the default protocol: 95 °C for 20 s, 40 cycles of 95 °C for 1 s and 60 °C for 20 s. The number of cycles necessary for the fluorescent intensity to increase exponentially, C_t_ values, were recorded and normalized to β-actin expression. The comparative ΔC_t_ method [35] was used to analyze the mRNA expression levels compared to undifferentiated Sim1-Puro ESCs. Fold differences in relative mRNA expression levels over the control cultures are reported for each gene (n ≥3 for all groups).

**Table 2:** TaqMan Gene Expression Assays for qRT-PCR

Marker	Life tech identification
Bassoon	Mm00464452_m1
Beta-Actin	Mm00607939_S1
Psd95	Mm00492193_m1
SV2a	Mm00491537_m1
VGluT2	Mm00499876_m1

### Electrophysiology

Whole-cell electrodes had an open tip resistance of 2–6 MOhms when filled with K- or Cs-glucuronate internal solutions (in mM, all from Sigma): 140 K-glucuronate 10 NaCl, 5 MgCl_2_, 0.2 EGTA, 10 HEPES, pH adjusted to 7.4 with KOH; or, 130 Cs-glucuronate, 5 MgCl_2_, 0.2 EGTA, 10 HEPES, pH adjusted to 7.4 with CsOH. Both internal solutions were supplemented with 5 mM Na-ATP and 1 mM Na-GTP. Culture dishes were perfused at approximately 1 mL/min with Tyrode’s solution (in mM): 150 NaCl, 4 KCl, 2 MgCl_2_, 2 CaCl_2_, 10 glucose, 10 HEPES, pH adjusted to 7.4 with NaOH. Currents and membrane potentials were recorded with Axopatch 200 amplifiers (Molecular Devices, Sunnyvale, CA), filtered at 1 kHz and digitized at 10 kHz using pClamp 9.2 (Molecular Devices, Sunnyvale, CA). Tetrodotoxin (0.5 μM), tetraethylammonium (30 mM), and 4-aminopyridine (5 mM) were dissolved in Tyrode’s solution. Agonists were dissolved at 100 μM in 160 mM NaCl, 2 mM CaCl_2_, 10 mM HEPES, pH adjusted to 7.4 with NaOH. Drug solutions were applied by local perfusion from a multi-barreled delivery pipette [6].

### Statistical analysis

Three biological replicates of each condition were performed. Three sample pictures were analyzed from each replicate for cell counting. Statistical analysis was performed in Statistica software (version 5.5; StatSoft, Tulsa, OK). Unless otherwise stated, multiple comparison statistics were accomplished using Scheffe’s post hoc test for analysis of variance (ANOVA) with a 95 % confidence level. Values are reported as the mean plus or minus standard deviation.

## Results and discussion

While ESCs provide a plentiful cell source to better study a variety of cell types, the heterogeneity of cells induced from ESC cultures by current protocols retains the problems of dissection and isolation. Recently, our lab reported a MN line where previously established Hb9 enhancer regions were used to drive expression PAC [32]. Unfortunately, a highly conserved efficient enhancer region for Sim1 has not been identified [36]. Thus, random insertion of a Sim1 enhancer-promoter driving PAC was not achievable. Homologous recombination has been used ubiquitously to generate knockout animals and cell lines [33]. Previously, our lab reported an Olig2-Puro cell line that has PAC knocked into the Olig2 locus, enabling the Olig2 gene regulatory elements to control PAC expression [21]. This method was adopted for the Sim1 locus, such that transgenic mouse ESCs were generated by the electroporation of a vector containing the PAC gene into the Sim1 locus. The use of Cas9/CRISPR technology greatly increased the efficiency of recombination by inducing a double strained break in the first exon of Sim1. This paper describes a method to overcome the heterogeneity of ESC-derived V3 IN cultures using this Sim1-Puro ESC line. The generated cell line would not be used clinically, but would serve as a tool for furthering understanding of V3 INs through in vitro and in vivo studies in rodent models of spinal cord injury.

### Targeted PAC insertion into the Sim1 locus

The Sim1-Puro cell line was generated by using a targeting vector to insert a resistance cassette into the open reading frame of the Sim1 gene. Two homology arms approximately 2 kb and 10 kb in size were inserted into the targeting vector flanking the resistance cassette (Fig. 1a,b). RW4 ESCs were electroporated with the targeting vector and a set of Sim1 targeting Cas9/CRISPR plasmids. The expected homologous recombination event is illustrated in Fig. 1c. JPCR with one primer hybridizing to genomic DNA outside of the homology arms and one primer hybridizing within the resistance cassette was used to screen for insertion of the resistance cassette into the Sim1 locus (Fig. 1d,e).

While Sim1-Cre heterozygous animals have been reported with appropriate neuronal migration, in Sim1 knockout mice, the neurons fail to properly migrate [37]. Furthermore, Sim1^–/–^ animals are not viable [37], further indicating the importance of keeping at least one allele of Sim1 intact. Thus, it was important that only one allele had the puromycin gene knocked into the Sim1 locus. Two clones (6E and 8E) that screened positive for insertion of PAC by JPCR were analyzed with a copy number assay, which reported values of approximately 1 for both clones (Fig. 1f), indicating that only one copy of the PAC gene was inserted into the cells, and that the other Sim1 allele did not have PAC knocked in. The appropriate JPCR results coupled with the copy number assay results together indicate that one copy of PAC was successfully inserted into the Sim1 locus, resulting in the desired Sim1-Puro cell line. The resulting transgenic-ES cell line (Sim1-Puro Clone 6E) was used for all subsequent studies.

### Increased purity of Sim1^+^ cells after puromycin selection

To test for PAC expression in Sim1^+^ cells, RW4 and Sim1-Puro EBs were subjected to induction and selection (2^−^/6^+^/1p) protocols as illustrated in Fig. 2a. Visual assessment of calcein-AM staining of selected and unselected cultures showed that 2 μg/mL puromycin was sufficient to kill all RW4 cells. The unselected RW4 culture (Fig. 2b) looked healthy and confluent, while the selected RW4s had no remaining live cells (Fig. 2c). In the Sim1-Puro selected and unselected cultures, the unselected cultures contained many cells of non-neuronal morphology, while the selected culture contained fewer cells with non-neuronal morphology (Fig. 2d,e). The efficacy of RW4 selection indicated that the 2 μg/mL puromycin concentration had sufficient potency to remove all non-PAC-expressing cells but allowed survival of PAC-expressing cells, whereas 4 μg/mL puromycin resulted in very low cell viability (data not shown). Thus, 2 μg/mL puromycin was used for subsequent studies.

**Fig. 3:**
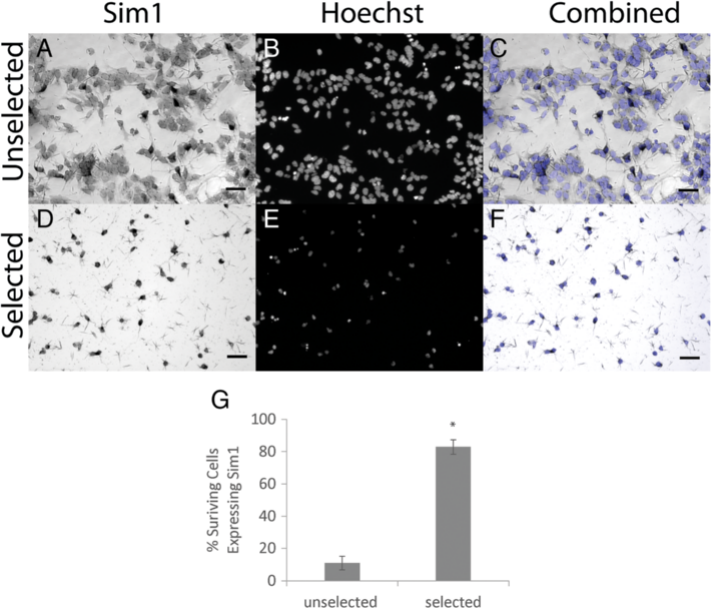
Puromycin selection increases the percentage of cells expressing Sim1 in Sim1-Puro cultures. **a**–**c** Sim1 in situ hybridization (*dark cell bodies*) and nuclear staining (*blue*) on unselected cells. **d**–**f** Sim1 in situ hybridization and nuclear staining on selected cells. **a** & **d** Sim1 in situ hybridization. **b** & **e** Nuclear marker Hoechst (*blue*). **c** & **f** Overlaid images. **g** Selected cultures enriches for percentage of Sim1^+^ cells. * Denotes *P* <0.05 compared to unselected group

Due to the lack of a specific Sim1 antibody, the purity of cultures post-selection was assessed by performing in situ hybridization on dissociated cultures. The percentage of Sim1^+^ cells increased significantly from 11 % in unselected cultures to 83 % after selection (Fig. 3). This increase indicated that puromycin selection of the Sim1-Puro line successfully enriched for Sim1^+^ cells. Furthermore, the presence of Sim1^+^ cells via in situ hybridization corroborates the copy number assay data and suggests that the Sim1 gene on the non-altered allele should be functional, at least to the properly spliced mRNA stage. In addition to looking for Sim1^+^ cells, we also assessed the selected and unselected cultures for Uncx (a V3 marker) and Nkx2.2 (a p3 marker) expression. As seen in Additional file 2: Figure S2, we saw an increase in the percentage of cells expressing Uncx (from 20.1 ± 1.7 in the unselected culture to 43.8 ± 4.1 in the selected culture) and no change in the percentage of cells expressing Nkx2.2. This also points to an enrichment of V3 INs in the selected culture.

To assess what other cell types were present after selection, cultures were stained with Hb9, Isl1, Ki67, A2B5, and nestin (Additional file 2: Figure S2). Compared to the unselected cultures, the selected cultures showed a decrease in non-V3 lineage markers. These results indicate that the cells surviving selection contained predominantly the desired cell type.

Based on the in situ hybridization and immunocytochemistry results, the Sim1-Puro cell line is able to enrich a culture for Sim1^+^ cells, such that most of the cells in a selected culture are Sim1^+^. The purity of the selected Sim1-Puro line is lower compared to the 99 % purity of MNs reported from the Hb9-Puro line previously generated in our lab [32]. One reason for lower purity is that the Hb9-Puro line was assessed with an antibody while the Sim1-Puro line was assessed with in situ hybridization – an mRNA based technique. Because mRNA is transient and expressed prior to protein synthesis, a portion of the surviving cells could be Sim1 mRNA-negative but have recently expressed Sim1 mRNA and thus still have Sim1 and PAC protein present. They would not be Sim1^+^ by in situ hybridization, but would still be V3 INs and might stain positive with a Sim1 antibody, if one existed. The immunocytochemistry data for MNs and glia showed a scarcity in the MN population and some glia population, suggesting that the selected culture consists mostly of V3 INs. Furthermore, the increase of the percentage of cells staining positive for Uncx, from 20.1 % of cells in unselected cultures to 43.8 % of cells in the selected cultures, showed that V3 INs were indeed enriched. The disparity between the percentage of cells expressing Uncx protein (43.8 %) versus Sim1 mRNA (82.8 %) could be due to variations of the Sim1 and Uncx expression time course or mRNA being expressed at earlier time points versus protein. Furthermore, the combination of percent selected cells expressing Uncx (43.8 %) and Nkx2.2 (31.6 %) did sum to be much closer to the percentage of Sim1^+^ cells, suggesting that Uncx may be expressed slightly later than Sim1 and that cells lacking Uncx expression are still Nkx2.2 positive. Unfortunately, because the Uncx and Nkx2.2 antibodies are both mouse IgG antibodies, we were unable to co-stain with these two markers.

Another reason for having a lower percentage of Sim1^+^ cells is that, compared to the Hb9-Puro study, a lower puromycin concentration was used in this study for selection. Previously, 4 μg/mL puromycin was used for the Hb9-Puro cell line [32] and 2 μg/mL was used here with the Sim1-Puro cell line since less than 100 cells/cm^2^ were observed after 4 μg/mL puromycin selection (versus 50,000–100,000 cells/cm^2^ for 2 μg/mL puromycin). It was previously reported that Hb9 mRNA levels increase approximately 400-fold versus uninduced controls (no RA and no SAG) after MN induction, whereas Sim1 mRNA levels only increase approximately 100-fold after induction [7, 21], suggesting that Sim1 is not as strongly expressed as Hb9. This fold change difference can be partially attributed to the lower induction protocol efficiency (~60 % for MNs vs. ~10 % for V3 INs), but it could also be due to Sim1 gene regulatory elements driving less robust expression than those for Hb9. Lower levels of PAC expression in each cell could result in increased sensitivity at a lower puromycin concentration. The relatively low purity of the selected Sim1-Puro cell line could be a problem for longer term studies. While we were able to observe distinct neuronal morphology up to 2 weeks post-selection, the presence of proliferation marker Ki-67 in our culture indicates that, in time, glia may overwhelm V3 INs. Furthermore, glia survival will limit the control we have in defining cell ratios of seeded cell types in any co-cultures or in in vivo transplantation studies moving forward. While the resulting cultures are not 100 % Sim1^+^ cells, the observed enrichment is sufficient to allow for further studies of ESC-induced V3 INs.

### Selected cultures exhibit neuronal markers and achieve functional maturity

Glial-conditioned media was needed for the long-term survival of selected Sim1-Puro V3 INs. Initially, without conditioned media, we observed axonal degeneration by 7 days in culture and ultimately cell death. We hypothesized that providing glial signaling cues would aid in V3 IN survival. Thus, the P-Olig2 ESC line, where the PAC gene was incorporated into the Olig2 locus was used to obtain glia and to produce conditioned media. Selection of P-Olig2 cells results in both MNs and glia (oligodendrocytes and astrocytes) [21]. While this was not ideal for generating a high purity MN population, the resulting pMN population generated glial-conditioned media suitable for long-term cultures of V3 INs. Using this conditioned media, V3 IN cultures survived for more than 14 days, which was necessary for maturation and synapse formation to be observed.

**Fig. 4:**
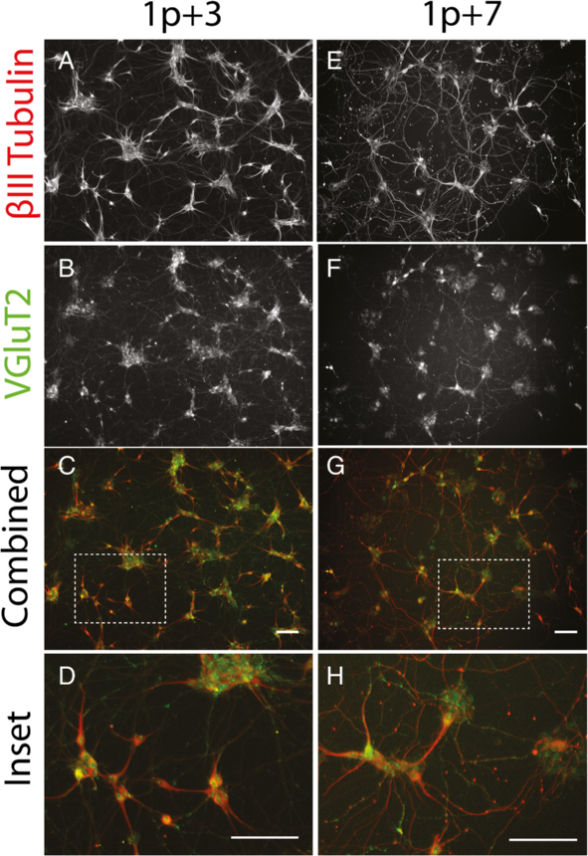
Sim1-Puro cultures exhibit glutamatergic and neuronal markers through the first week post-selection. **a**–**d** Staining 3 days post-selection. **e**–**h** Staining 7 days post-selection. Immunocytochemistry marker labeled on far left. **a** & **e** β-III tubulin (*red in combined*); **b** & **f** VGluT2 (*green in combined*); **c** & **g** Combined image; **d** & **h** Zoomed in combined image. Scale bar = 100 μm

To assess the phenotype selected cultures, immunocytochemistry was performed. β-III tubulin was used to verify the neuronal identity of the selected cells. As seen in Figure 4a and e, selected cells express β-III tubulin with axonal morphology at both 3 and 7 days post-selection (1p +3 and 1p +7). VGluT2, a vesicular glutamate transporter found in synaptic vesicles at presynaptic nerve terminals of excitatory neurons, was used as a marker of glutamatergic neurons. As seen in Figure 4b and f, selected cells exhibited punctate VGluT2 staining along their axons at 3 and 7 days post-selection. The VGluT2 and β-III tubulin staining aligned well (Fig. 4c,d), and the VGluT2 puncta are clearly visible in the zoomed in insets (Fig. 4g,h). These two antibodies verify that the selected cells are indeed glutamatergic neurons. To characterize the 1p +7 cultures further, proliferation marker Ki-67 and oligodendrocyte marker O4 were quantified along with β-III tubulin as a percentage of total cells (Additional file 2: Figure S2C). Only a few percent of the cells expressed Ki-67 and O4, suggesting that glia overtake of the culture did not occur.

Because these images were taken 4 days apart, some disparities between the left and right columns of Figure 4 were expected. We expected to observe maturation of axonal processes (longer axons and more connectivity between each other), which can be observed in Figure 4g. Furthermore, at 7 days post-selection, the cells are more sparsely distributed. This is not unexpected due to pruning observed in neuronal development. While some debate exists in the literature over whether INs undergo apoptosis as observed in MNs, it has been shown that ventral spinal INs do undergo apoptosis after they become post-mitotic [37, 38]. This occurs generally between e14 and P0 [37]. Our induction ends (1p +0) when Sim1 is strongly expressed, roughly equivalent to e11.5 [39], so apoptosis would be expected in the ensuing week. Taken together, the differences between the left and right columns of Figure 4 are consistent with expected neuronal maturation.

**Fig. 5:**
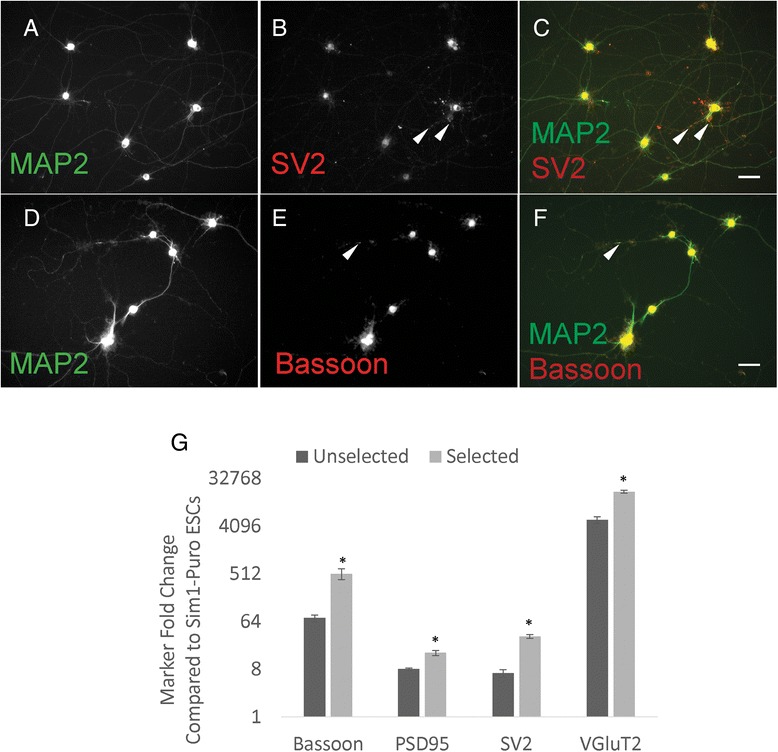
Sim1-Puro cultures exhibit synaptic marker protein and mRNA expression after 2 weeks. **a**–**f** Two-week selected cultures stained with dendritic marker MAP2 and synaptic markers, SV2 and Bassoon. Synaptic markers (**b** and **e**, *red*) exhibit puncta that overlap with MAP2 (**c** and **f**, *white arrows*) suggesting synapse formation. Scale bar = 50 μm. **g** Two-week selected cultures express increased synaptic marker (Bassoon, PSD95, and SV2) and VGluT2 (glutamatergic neuron) mRNA levels compared to unselected cultures (normalization control – Sim1-Puro ESCs). * Denotes *P* <0.05 compared to the unselected group

To determine whether selected V3 INs are maturing, immunocytochemistry was performed on cultures 2 weeks post-selection. MAP2, a dendritic marker, was used to identify neurons in culture. SV2 and Bassoon staining shows positive, punctate, synaptic marker staining along axons, indicating potential synapse formation (Fig. 5a–f). The presence of positive synaptic marker staining within the cell body was not expected. We hypothesized that if the synaptic marker mRNA was still being expressed, protein translation could be in progress and result in positive staining of the cell body. Thus, qRT-PCR was performed on 1p +14 (2 weeks post-selection) to assess expression levels of synaptic markers. Synaptic markers SV2, Bassoon, and PSD95, as well as glutamatergic neuron marker, VGluT2, were assessed in selected and unselected cultures. The resulting qRT-PCR data shows an upregulation of all markers in all conditions compared to the ESC controls and an increase in all marker expression between selected and unselected cells (Fig. 5g). These results must be considered within the context of a population-averaged assay. While the results suggest that there are more cells expressing synaptic markers in selected versus unselected cultures, the data only indicate that in selected cultures there is a greater percentage of neurons than in unselected ones. This is due to normalization of the data to the internal control β-actin, effectively normalizing expression levels to cell count. Additionally, the relative low levels of synaptic marker expression suggest that neurons within the cultures at 2 weeks are working towards but have not yet formed mature synapses.

### Electrophysiology

**Fig. 6:**
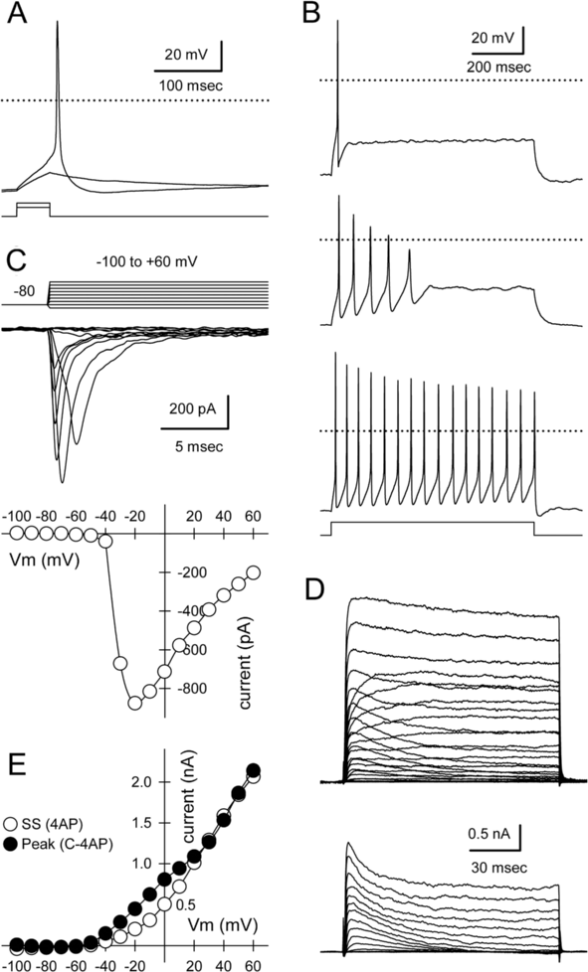
Sim1-Puro selected cultures exhibit action potential firing and voltage gated currents consistent with neuronal maturation. **a** Sub- and supra-threshold voltage responses recorded under current clamp in a 1p +2 cell with 50 msec square pulse injection (6 and 8 pA). **b** Examples of cells firing single (1p +11), adapting (1p +11), and multiple (1p +10) action potentials during an 800 msec depolarizing pulse (44 pA). **c** Currents mediated by tetrodotoxin-sensitive sodium channels evoked by steps from −100 to +60 mV from a holding potential of −80 mV (below). Peak inward current plotted as a function of step potential (1p +13; Cs-glucuronate internal solution). **d** Outward currents mediated by voltage-gated potassium channels for steps from −100 to +60 mV from a holding potential of −80 mV recorded in 0.5 μM tetrodotoxin (TTX) alone and in TTX plus 5 mM 4-aminopyrridine (4AP) (below). 4AP-sensitive currents revealed by subtracting current in TTX and 4AP from current in TTX alone (1p +8, K-glucuronate internal solution). **e** Current–voltage relation for steady-state current recorded in TTX plus 4AP (*open symbols*) and for peak 4AP-sensitive current (*filled symbols*)

In addition to using immunocytochemistry to characterize the Sim1-Puro line, electrophysiological recordings of the selected cells were also used to assess maturation. Whole-cell electrodes were used to test for action potential generation under current clamp and to record voltage- and ligand-gated currents under voltage clamp. Recordings were obtained from more than 50 cells between 2 and 5 days after puromycin selection (1p +2 to 1p +5; early) and more than 60 additional cells between 6 and 13 days post-selection (1p +6 to 1p +13; late). Cell capacitance increased and input resistance decreased with time after selection consistent with an increase in cell size (Table 3). Resting membrane potential (V_rest_) became more negative with time as did the proportion of cells that maintained V_rest_ less than −50 mV without the need for DC hyperpolarization. As shown in Figure 6, depolarizing current injection elicited action potentials in cells as early as 2 days after puromycin selection. With DC hyperpolarization to −60 mV and long duration (800 msec) depolarizing current pulses, all 14 of the early (1p +2 to 1p +5) cells tested were able to fire multiple action potentials, 57 % showed prominent spike adaptation, and the remaining 43 % fired repetitively with little adaptation. Most late (1p +6 to 1p +13) cells (70 %) also fired multiple spikes; however, 30 % only fired single action potentials. For cells that produced multiple spikes the firing properties (Table 3) were more similar to the ventral than the dorsal V3 interneuron population as characterized in mouse spinal cord slices [14, 40].

**Table 3:** Electrophysiological properties of Sim1-Puro selected cells

Parameter	1p +2–5	1p +6–13
Capacitance (pF)	18.3 ± 1.3 (51)	24.2 ± 0.9 (68)^a^
Input Resistance (GOhm)	1.64 ± 0.49 (51)	0.96 ± 0.17 (68)^a^
V rest (mV)	−28.5 ± 2.1 (43)	−43.7 ± 1.2 (64)^a^
% V rest less than −50 mV	5 %	28 %^a^
Rheobase (pA)	10.6 ± 2.3 (14)	18.3 ± 2.7 (34)
1^st^ spike latency (msec)	214 ± 43 (14)	153 ± 19 (24)
1^st^ spike amplitude (mV)	84 ± 4.5 (14)	95 ± 3.5 (24)
absolute amplitude (mV)	23 ± 4.1 (14)	34 ± 3.5 (24)
1^st^ spike threshold (mv)	−32.4 ± 0.8 (14)	−35.1 ± 1.0 (24)
1^st^ spike width (msec)	4.4 ± 0.5 (14)	2.9 ± 0.3 (24)^a^
10 Hz 1^st^ latency (msec)	44 ± 3.2 (14)	37 ± 2.0 (24)
10 Hz 1^st^ frequency (Hz)	11 ± 0.3 (14)	12 ± 0.3 (24)^a^
10 Hz frequency adaptation	1.2 ± 0.04 (14)	1.3 ± 0.05 (24)
10 Hz after potential (mv)	0.9 ± 0.5 (14)	−0.9 ± 0.7 (24)
*f-I* slope 1^st^ interval	0.59 ± 0.07 (14)	0.44 ± 0.03 (24)
*f-I* slope average frequency	0.54 ± 0.07 (14)	0.36 ± 0.03 (24)
Sag at −90 mV (mV)	3.3 ± 1.1 (14)	3.3 ± 0.7 (24)
Peak I Na (nA)	−1.06 ± 0.12 (40)	−2.09 ± 0.16 (58)^a^
Vm for peak I Na (mV)	−21.0 ± 1.6 (40)	−26.1 ± 1.4 (58)^a^

Values presented as mean ± SEM (number of cells). ^a^Denotes significantly different from 1p +2–5 days by *t*-test, Mann–Whitney rank sum test, or Z-test. Cell capacitance and input resistance were determined from 10 mV voltage clamp steps from a holding potential of −80 mV. First spike latency, amplitude, absolute amplitude, threshold and half-width were determined for the first spike recorded at threshold depolarization. In addition, 800 msec depolarizations that elicited spiking with an average frequency of 10 Hz were used to measure 1^st^ latency, instantaneous frequency from the first inter-spike interval (ISI), frequency adaptation (ratio of last to first ISI), after potential as well as the initial slope of frequency versus current (*f-I*) plots of instantaneous and average frequency. Sag in voltage responses was determined for 800 msec hyperpolarizing current injections from −60 mV

Under voltage clamp, Sim1-Puro-selected cells exhibited fast transient inward current and slow rising transient and sustained outward currents with depolarizing voltage steps from a holding potential of −80 mV (Fig. 6). Inward current was blocked by the selective sodium channel antagonist, tetrodotoxin (0.5 μM). Outward current was reduced in cells filled with the potassium channel blocker cesium, or by extracellular exposure to the organic potassium channel blockers, tetraethylammonium (30 mM) and 4-aminopyrridine (5 mM). As observed in other cell types [41], tetraethylammonium inhibited sustained outward currents, while 4-aminopyrridine reduced transient outward current. These results indicate the Sim1-Puro cells are expressing functional ion channels and behave in an appropriate manner for glutamatergic neurons.

Because mature V3 interneurons in acute slice recordings fire multiple spikes [14], we evaluated the relationship between spiking phenotype and a number of physiological parameters (Additional file 3: Figure S3). Cells from days 6–13 that fired single action potentials had a significantly higher rheobase than adapting or multiple spiking cells. In addition, single-spiking cells had the lowest mean ratio of inward to outward current, although this difference was not significant. Taken together, the results in Additional file 3: Figure S3 suggest that adapting and single spiking cells exhibit only minor differences in their physiological properties, which may be due to the in vitro environment in dissociated cell culture or may reflect a bona fide difference between ESC-derived and native neurons.

**Fig. 7:**
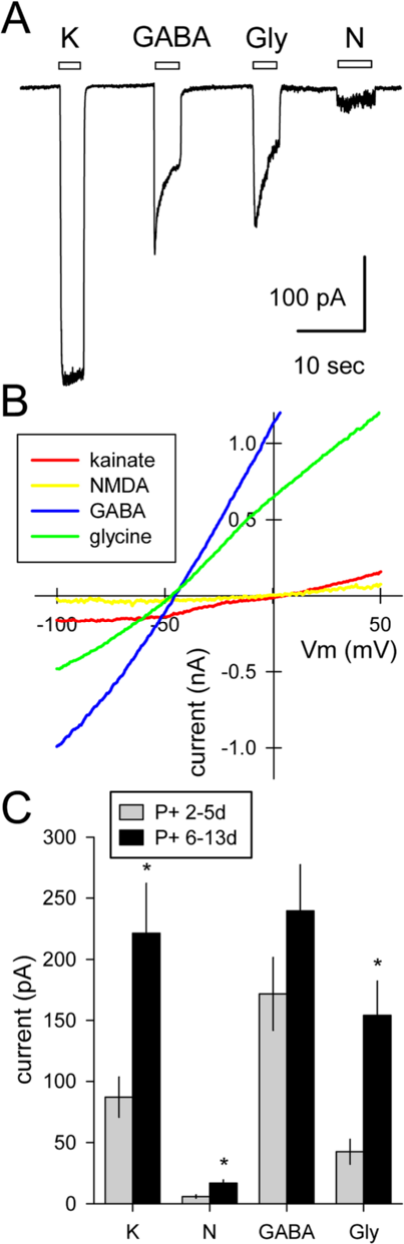
Excitatory and inhibitory agonists activate appropriate whole-cell currents in Sim1-Puro selected cultures. **a** Whole-cell currents evoked by kainate (K), γ-aminobutyric acid (GABA), glycine (Gly), and N-methyl-D-aspartate (NMDA, N; with 1 μM added glycine). Holding potential, −80 mV. **b** Agonist-evoked currents recorded during voltage ramps from −100 to +50 mV at 1.2 mV/msec. GABA and Gly evoked a current that reversed polarity at −48.6 ± 2.0 mV (n = 21) and −47.7 ± 2.2 mV (n = 10), respectively, consistent with activation of channels selective for chloride. K and NMDA evoked currents reversed at −1.4 ± 5.4 mV (n = 24) and −5.3 ± 7.2 mV (n = 4), respectively, consistent with activation of cation selective channels. **c** Currents increased with time in culture after puromycin selection (n = 30 to 45 cells per bar). *Denotes *P* <0.01 (Mann–Whitney rank sum test)

Selected Sim1-Puro cells expressed a number of neurotransmitter-gated channels, as determined by exposure to the inhibitory transmitters γ-aminobutyric acid (GABA) and glycine, as well as excitatory agonists for α-amino-3-hydroxy-5-methyl-4-isoxazolepropionic acid (AMPA)/kainate and N-methyl-D-aspartate (NMDA) receptors. Agonists were applied at 100 μM, and the NMDA solution was supplemented with 1 μM glycine, which is required as a co-agonist. At a fixed holding potential of −80 mV, all four agonists evoked inward currents (Fig. 7a). When the holding voltage was ramped from negative to positive potentials, the currents reversed polarity consistent with the ionic selectivity of their underlying ion channels. Current evoked by GABA or glycine reversed near −50 mV as expected for chloride selective channels, whereas kainate and NMDA evoked currents that reversed near zero mV, consistent with selective permeability to cations. These results indicate the Sim1-Puro cells are expressing appropriate neurotransmitter receptors.

The data presented indicates that selected cells exhibited maturation by decreasing input resistance, increasing membrane capacitance, and acquiring more negative resting potentials. Inward and outward currents, appropriate agonist and blocker responses, and spike firing and adaptation also point to the in vitro maturation of the V3 INs. Additionally, the data presented within Table 3 suggests similar trends as the values reported in Borowska et al. [14, 40], with the more mature Sim1-Puro V3 INs having capacitance and input resistance values that fall fairly close to the error range previously reported for the ventral V3 IN population. A few differences in the cells recorded in our study deserve mention. Firstly, the ESC-derived V3 population is likely to exhibit more rostral positional identity than the lumbar populations studied by Borowska et al. [40]. The use of low RA to derive V3 INs will result in a more rostral phenotype [7] than the lumbar population analyzed in earlier studies [14, 40]. Secondly, the values reported in the literature for V3 INs were determined by recordings from slices and not from neurons in dissociated cell culture*.* The difference in environment may underlie modest differences between our results and literature reported data. Not only do the ESC-derived V3 INs in vitro lack appropriate cues for migration, they are also deprived of physiologically normal pre- and post-synaptic connections. Thus, the slightly different than slice recording measurements observed in isolated ESC-derived V3 INs are not unreasonable.

**Fig. 8:**
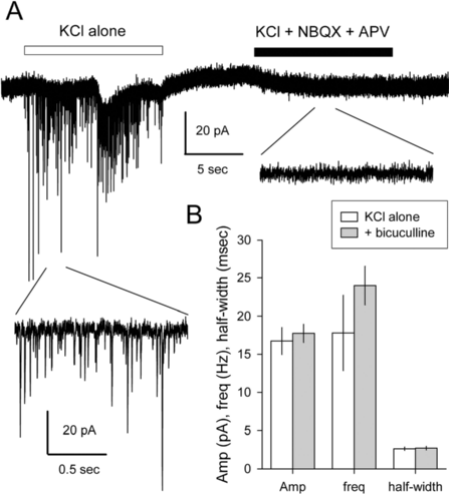
Sim1-Puro-selected cultures present synaptic currents consistent with glutamatergic neurons. **a** Whole-cell current recorded at −80 mV during exposure to Tyrode’s solution containing elevated potassium (10 mM; KCl alone) as indicated by the open box, or 10 mM potassium Tyrode’s that also included 30 μM NBQX and 50 μM APV to block AMPA/kainate and NMDA receptors, respectively, as indicated by the filled box. Short segments during each exposure are shown on a 10-fold expanded time scale. **b** Plots of mean ± sem amplitude (Amp, pA), frequency (freq, Hz), and width at half amplitude (half-width, msec) of synaptic events evoked during exposure to elevated KCl alone (*open bars*, four cells) or with 200 μM of the GABA antagonist bicuculline methiodide (*grey bars*, three cells)

In addition to currents evoked by exposure to exogenous agonists, some of the selected Sim1-Puro cells displayed spontaneous inward currents that resembled excitatory postsynaptic currents observed in primary neuronal culture (Fig. 8). The frequency of spontaneous events increased substantially during local perfusion with elevated KCl (10–20 mM) to depolarize presynaptic terminals. Consistent with the glutamatergic phenotype of V3 interneurons, spontaneous currents in selected Sim1-Puro cultures were unaffected by the GABA_A_ receptor antagonist, bicuculline (200 μM), but were eliminated during superfusion with a combination of NBQX (30 μM) and APV (50 μM), glutamate receptor antagonists that block AMPA/kainate and NMDA receptors, respectively [42]. These observations of spontaneous inward currents resembling postsynaptic currents functionally confirms the glutamatergic phenotype expected for V3 INs. Furthermore, these recordings at 1p +10 confirms the qRT-PCR data and indicates that selected Sim1-Puro V3 INs are able to mature into synapsing glutamatergic neurons.

## Conclusion

In this study, we have demonstrated that PAC was successfully knocked into the Sim1 locus of a mouse ESC line. The resulting cell line allows for the enrichment of Sim1^+^ cells post-induction. The selected population exhibits characteristics consistent with what is expected of V3 INs at this stage of development. This novel cell line allows for the further understanding of an understudied population of spinal INs. It is, however, important to keep in mind the limitations of this generated cell line. First of all, this cell line is limited by the robustness of the induction protocol. A low puromycin concentration is used in the selection process to help improve V3 IN yields after selection. This low puromycin concentration resulted in a selected culture that is not 100 % pure. Secondly, due to difficulties in obtaining primary cultures, we have not shown how our selected V3 IN cultures compare to primary cells in vitro. Finally, the generated cell line is not designed for use in a clinical setting due to its transgenic and murine nature. However, the Sim1-Puro cell line could be useful in understanding IN maturation and CPG formation. Co-culturing Hb9-Puro MNs and Sim1-Puro V3 INs could be a starting point for a bottom-up approach to understanding CPG circuitry and ultimately designing novel therapeutics for spinal cord injury.

## Additional files

Additional file 1: Figure S1. Generation of the Sim1-Puro-pStartTK targeting vector. 1) Homologous recombination: The backbone was a Gateway-compatible plasmid, pStartK was amplified by PCR and Sim1 homology arms were incorporated into pStartK from RP23-223 M2 BAC using red recombinase competent bacteria. 2) Homologous recombination: Similar to step 1, a chloramphenicol resistance gene flanked by AscI cut sites from pkD3 was inserted into the open reading frame of the Sim1 gene by red recombinase competent bacteria. 3) Digest and ligate: The chloramphenicol resistance gene was then replaced via restriction enzyme digestion and ligation by a dual resistance cassette consisting of, from 5’ to 3’: Asc1 cut site, PAC gene, floxed phosphoglycerate kinase I promoter driving neomycin phosphotransferase (PGK-neo) cassette, and AscI site. 4) Gateway recombination: A negative selection thymidine kinase gene was incorporated into the finished Sim1-Puro-pStartTK vector using pWS-TK3 plasmid and Gateway LR clonase II kit. AMP, Ampicillin resistance gene; AscI, Restriction enzyme site; attB1 & attB2, Gateway recombination results; CAT, Chloramphenicol resistance; HA, Homology arm; JPCR, Junction PCR; KAN, Kanamycin resistance; ori, Origin of replication; PAC, Puromycin resistance gene; PGK, Phosphoglycerate kinase promoter sequence; Neo, Neomycin resistance gene; Sim1 ATG, Translation start in Sim1 Exon1; TK, Thymidine kinase. (TIF 364 kb) Additional file 2: Figure S2. Identification of non-Sim^+^ populations post-selection. (**A**) Quantification of selected and unselected Sim1-Puro cultures at 1p +0 stained with Uncx, Nkx2.2, Ki-67, Isl1, and Hb9 at end of selection. * Denotes *P* <0.05 compared to unselected group. (**B**) Quantification of selected cultures at 1p +0 stained with A2B5 and nestin at end of selection. (**C**) Quantification of selected cultures at 1p +7 stained with β-III tubulin, Ki-67, and O4. (TIF 148 kb) Additional file 3: Figure S3. Physiological parameters associated with spiking phenotypes. (**A**) Rheobase (current required to reach threshold) plotted versus input resistance for cells from days 2–5 (squares) or days 6–13 (circles) that fired single (open), adapting (grey), or multiple (black) action potentials during an 800 msec current pulse. Input resistance decreased with time after selection, but did not correlate with spiking phenotype. Cells from days 6–13 firing single action potentials had a significantly higher rheobase (*P* <0.01, one-way ANOVA on ranks). (**B**) Steady-state outward current density (I K density) versus peak inward current density (I Na density) at 0 mV. Single-spiking cells had the lowest mean inward current density and the second highest outward current density but the differences were not significant. (**C**) Current density ratio (I Na/I K) versus threshold. Repetitive firing cells from days 6–13 had a significantly lower threshold (*P* <0.002, one-way ANOVA). Single-spiking cells had the lowest mean current density ratio, although the differences were not significant. (JPG 447 kb)

## Abbreviations

AMPA: α-Amino-3-hydroxy-5-methyl-4-isoxazolepropionic acid
CPG: Central pattern generator
DIG: Digoxigenin
EBs: Embryoid bodies
EDTA: Ethylenediaminetetraacetic acid
ESC: Embryonic stem cell
FACS: Fluorescence-activated cell sorting
GABA: γ-Aminobutyric acid
IN: Interneuron
MN: Motoneuron
NMDA: N-methyl-D-aspartate
PAC: Puromycin N-acetyl-transferase (puromycin resistance enzyme)
PBS: Phosphate buffered saline
PBT: PBS with 2 mg/mL bovine serum albumin and 0.1 % Triton X-100
PFA: Paraformaldehyde
qRT-PCR: Quantitative real time polymerase chain reaction
RA: Retinoic acid
SAG: Smoothened agonist
Shh: Sonic hedgehog
SSC: Sodium citrate buffer
