# Supplementary figures and images for: A puromycin selectable cell line for the enrichment of mouse embryonic stem cell-derived V3 interneurons
Source: Stem Cell Res Ther. 2015 Nov 10;6:220. doi: 10.1186/s13287-015-0213-z (PMC4641415; doi:10.1186/s13287-015-0213-z)

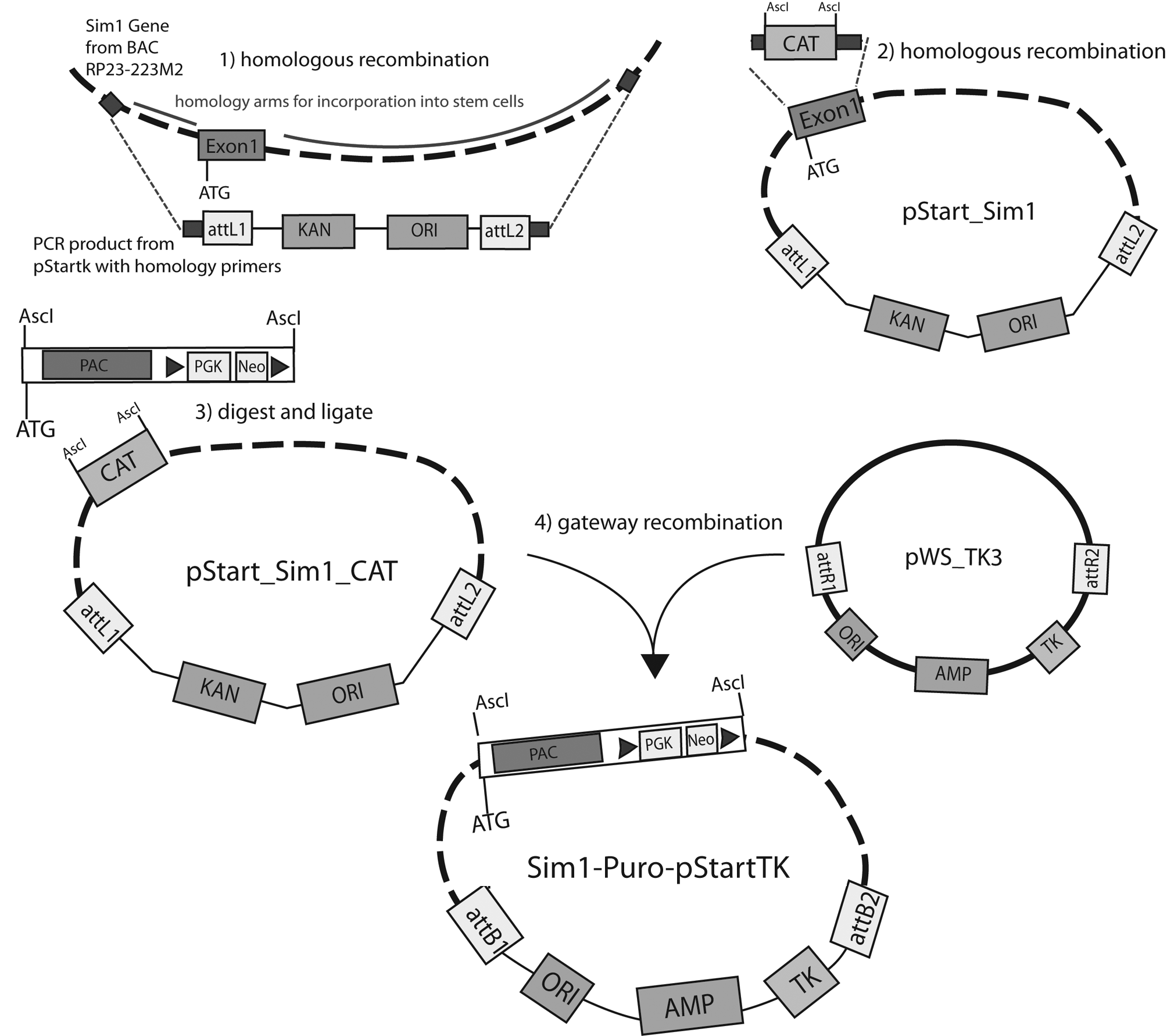

Supplement: Additional file 1: Figure S1. — Generation of the Sim1-Puro-pStartTK targeting vector. 1) Homologous recombination: The backbone was a Gateway-compatible plasmid, pStartK was amplified by PCR and Sim1 homology arms were incorporated into pStartK from RP23-223 M2 BAC using red recombinase competent bacteria. 2) Homologous recombination: Similar to step 1, a chloramphenicol resistance gene flanked by AscI cut sites from pkD3 was inserted into the open reading frame of the Sim1 gene by red recombinase competent bacteria. 3) Digest and ligate: The chloramphenicol resistance gene was then replaced via restriction enzyme digestion and ligation by a dual resistance cassette consisting of, from 5’ to 3’: Asc1 cut site, PAC gene, floxed phosphoglycerate kinase I promoter driving neomycin phosphotransferase (PGK-neo) cassette, and AscI site. 4) Gateway recombination: A negative selection thymidine kinase gene was incorporated into the finished Sim1-Puro-pStartTK vector using pWS-TK3 plasmid and Gateway LR clonase II kit. AMP, Ampicillin resistance gene; AscI, Restriction enzyme site; attB1 & attB2, Gateway recombination results; CAT, Chloramphenicol resistance; HA, Homology arm; JPCR, Junction PCR; KAN, Kanamycin resistance; ori, Origin of replication; PAC, Puromycin resistance gene; PGK, Phosphoglycerate kinase promoter sequence; Neo, Neomycin resistance gene; Sim1 ATG, Translation start in Sim1 Exon1; TK, Thymidine kinase. (TIF 364 kb) [file 13287_2015_213_MOESM1_ESM.tif]

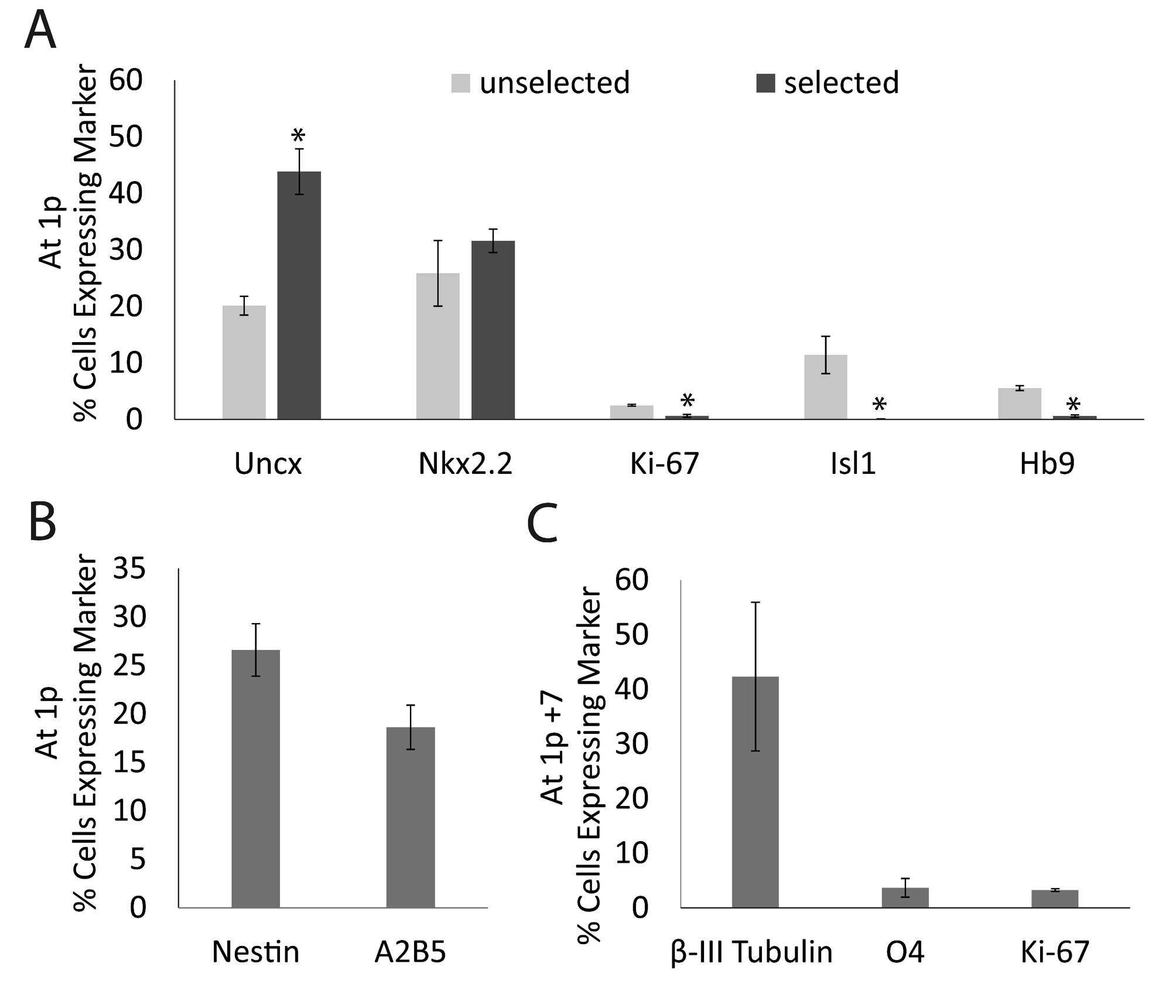

Supplement: Additional file 2: Figure S2. — Identification of non-Sim+ populations post-selection. (A) Quantification of selected and unselected Sim1-Puro cultures at 1p +0 stained with Uncx, Nkx2.2, Ki-67, Isl1, and Hb9 at end of selection. * Denotes P <0.05 compared to unselected group. (B) Quantification of selected cultures at 1p +0 stained with A2B5 and nestin at end of selection. (C) Quantification of selected cultures at 1p +7 stained with β-III tubulin, Ki-67, and O4. (TIF 148 kb) [file 13287_2015_213_MOESM2_ESM.tif]

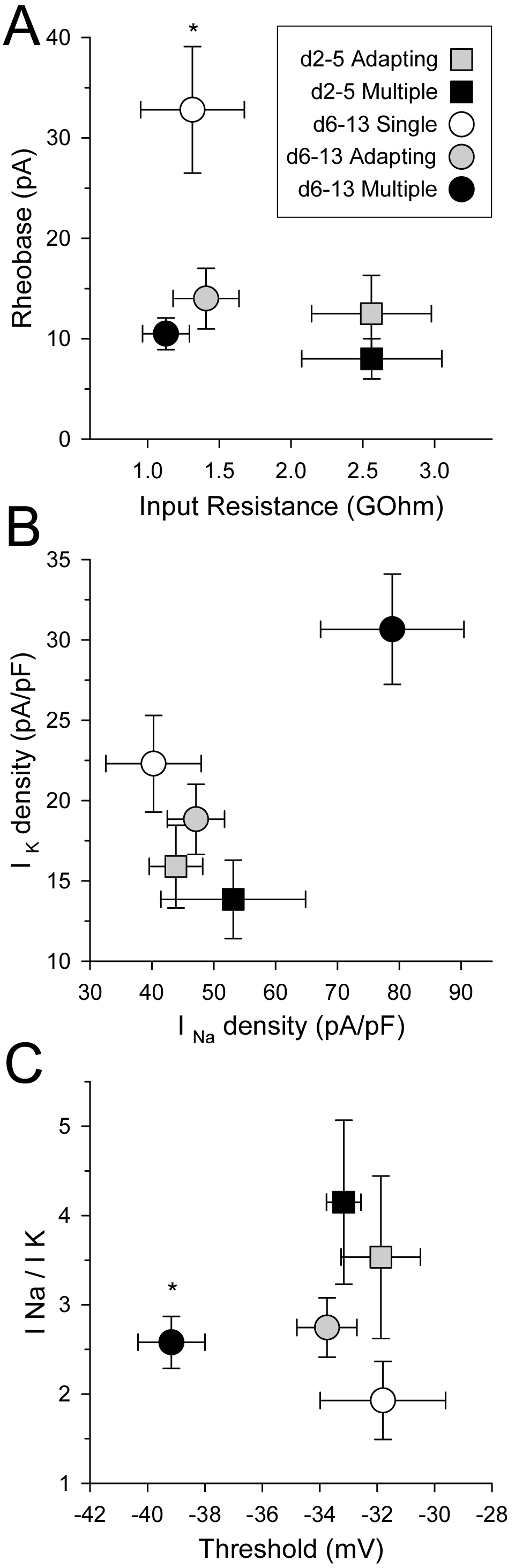

Supplement: Additional file 3: Figure S3. — Physiological parameters associated with spiking phenotypes. (A) Rheobase (current required to reach threshold) plotted versus input resistance for cells from days 2–5 (squares) or days 6–13 (circles) that fired single (open), adapting (grey), or multiple (black) action potentials during an 800 msec current pulse. Input resistance decreased with time after selection, but did not correlate with spiking phenotype. Cells from days 6–13 firing single action potentials had a significantly higher rheobase (P <0.01, one-way ANOVA on ranks). (B) Steady-state outward current density (I K density) versus peak inward current density (I Na density) at 0 mV. Single-spiking cells had the lowest mean inward current density and the second highest outward current density but the differences were not significant. (C) Current density ratio (I Na/I K) versus threshold. Repetitive firing cells from days 6–13 had a significantly lower threshold (P <0.002, one-way ANOVA). Single-spiking cells had the lowest mean current density ratio, although the differences were not significant. (JPG 447 kb) [file 13287_2015_213_MOESM3_ESM.jpg]
